# Parental autonomy support and future-oriented coping among high school students: Serial mediation of future time perspective and meaning in life

**DOI:** 10.3389/fpsyg.2022.895003

**Published:** 2022-08-03

**Authors:** Lianping Zeng, Xia Peng, Xiaoye Zeng, Hui Wang, Shifei Xiao, Yan Chen

**Affiliations:** ^1^School of Psychology, Guizhou Normal University, Guiyang, China; ^2^Zunyi No. 15 High School, Guiyang, China

**Keywords:** parental autonomy support, future time perspective, meaning in life, future-oriented coping, high school students

## Abstract

Guided by the ecosystem theory, this study aimed to explore the association between parental autonomy support and future-oriented coping of high school students, as well as the mediating effects of future time perspective and meaning in life in this relationship. A total of 707 Chinese high school students were involved in a paper questionnaire survey and data analysis. It was found that (1) parental autonomy support was significantly positively related to future-oriented coping. (2) Mediation analyses demonstrated that parental autonomy support directly affects future-oriented coping; parental autonomy support indirectly affects future-oriented coping through separate mediation and serial mediation of future time perspective and meaning in life. These findings have important implications for the improvement of future-oriented coping abilities of high school students.

## Introduction

Traditional coping (reactive coping) primarily focuses on past or existing stress events (Schwarzer and Taubert, 2002). However, a study found that individuals can predict future stressors and take measures in advance (Feng and Huang, 2002). This proactive coping to future events is called future-oriented coping (Gan et al., 2007; Feng et al., 2015), and it includes two forms, proactive coping and preventive coping, which have different motivations. Preventive coping is motivated by threat assessment and aims to minimize the loss in the future from uncertain stressors (e.g., failing in a final exam). Proactive coping is motivated by challenge assessment and aims to achieve future goals and promote self-improvement (Schwarzer and Taubert, 2002; Miao and Gan, 2018). Both forms are responses to future events in advance (Miao et al., 2017a). Previous studies have integrated the two forms into one variable, i.e., future-oriented coping (Feng et al., 2015; Miao and Gan, 2021). Thus, they are studied as a whole in this study. Previous research on future-oriented coping has focused on groups, such as the elderly, dysfunctional patients, and depressed patients. However, coping with potential threats and meeting future challenges are also important for high school students. Based on this, it is necessary to examine their future-oriented coping (Gan, 2011). At the same time, on the factors influencing future-oriented coping, previous research has focused on individual internal factors (e.g., personality traits, achievement motivation, reaction style, and expected emotion) (Wen et al., 2014; Feng et al., 2015; Próchniak and Próchniak, 2021), with insufficient attention paid to external environmental factors. For high school students, their parents are an important source of social support. A study showed that parental autonomy support enabled high school students to recognize their possible future selves and proactively accumulate resources to cope with potentially stressful events while achieving personal goals and self-improvement (Schwarzer and Luszczynska, 2008).

First, the influence mechanisms of parental autonomy support on future-oriented coping among high school students may be related to their future time perspective. Autonomous support from parents can lead high school students to actively focus on the future, thus incorporating future expectations into their cognition, attitudes, and behaviors, which can motivate them to pursue and achieve their goals (Miao et al., 2021). Second, the influence mechanisms may also be related to their subjective experience of meaning in life. As the basis and starting point for future planning, the meaning in life influences the individual's perception of goals and missions (Steger et al., 2008). Parental autonomy support encourages high school students to make decisions freely, which meets their current psychological developmental needs and thus enhances their sense of meaning in life. The experiential component (having meaning) and the motivational component (seeking meaning) of meaning in life help guide them to establish long-term goals and to work toward those goals.

## Parental autonomy support and future-oriented coping

As a new perspective in the field of coping, future-oriented coping is a more proactive and purposive way of coping. According to the ecosystem theory proposed by Bronfenbrenner (1986), the family has the most straightforward and profound influence on the individual. As a positive factor in the family, parental autonomy support refers to the emotional recognition and autonomous decision-making support that individuals receive from their parents (Ryan et al., 2015). It creates the conditions for high school students to grow independently and guides them to establish challenging goals (Weinstein et al., 2012). It also increases their confidence in controlling their surroundings and enables them to take a positive approach to prevent or cope with stressful events, i.e., enhancing future-oriented coping ability (Luo et al., 2014).

Previous research shows that parental autonomy support affects coping styles among high school students (Seiffge-Krenke and Pakalniskiene, 2011; Peng et al., 2020). However, the impact of parental autonomy support on future-oriented coping and its internal mechanisms remain to be explored. Thus, we formulated the following hypothesis:

*Hypothesis H1: Parental autonomy support positively affects future-oriented coping*.

## Future time perspective and meaning in life as the possible mediators

### The mediating role of future time perspective

Future time perspective is the individuals' awareness of future time and planning for future events. It can guide individuals' behavior through future goals and rewards (Zimbardo and Boyd, 1999; He et al., 2019). Social cognitive theory suggests that the environment indirectly affects individuals' behavior through cognitive factors (Bandura, 1986). It is inferred that the effect of parental autonomy support on future-oriented coping may arise by changing individuals' perceptions of future time. On the one hand, parental autonomy support can, in part, enhance personal growth initiatives, enabling individuals to think proactively about the future and improve their future time perspective. The important role of parental autonomy support in future time perspective was confirmed by the study of Cheng and Wang (2012). On the other hand, the cognitive-dynamic effect of future time perspective can motivate high school students to break through the time barrier by influencing their outcome expectations and value assessments. This enables them to accurately perceive potential future stressors and take timely action in response. Song et al. (2013) believed that individuals with future time perspectives have clearer and more explicit perceptions of future time, which can enhance individuals' expectations and motivation for future goals. It is inferred that parental autonomy support can enhance individuals' future-oriented coping ability by enhancing their future time perspective. Thus, we hypothesized the following:

*Hypothesis H2: Future time perspective mediates the relationship between parental autonomy support and future-oriented coping*.

### The mediating role of meaning in life

The meaning in life refers to the individual's perception of the meaning, value of the current life, the pursuit of the meaning, and purpose of the future life (Steger et al., 2008). It is an important guarantee for the psychological function of the individual to work normally (Yu and Deng, 2019). It includes two dimensions: the presence of meaning and the search for meaning. Presence of meaning means that the individual realizes that their life matters; the search for meaning concerns the extent to which individuals seek meaning in their lives (Steger et al., 2006). According to self-determination theory, perceived environmental support satisfies individuals' autonomy, competence, and relational needs, which contribute to maintaining individual internal and external motivation and fostering individual self-determined behavior (Ryan and Deci, 2000; Deci and Ryan, 2008; Liu et al., 2020) Parental autonomy support creates a supportive environment for high school students, which in part, meets their primary current needs and thus enhances their meaning in lives (Lambert et al., 2013). It has been shown that parental autonomy support is positively correlated with meaning in life (Luo and He, 2020; Shek et al., 2021). Furthermore, the increased meaning in life makes high school students actively focus on the future and proactively accumulate resources to cope with future stressors (Luo and He, 2020). Research has found that meaning in life plays an important role in enhancing individuals' future-oriented coping (Sougleris and Ranzijn, 2011; Miao et al., 2017b; Miao and Gan, 2021). Thus, the following hypothesis is offered:

*Hypothesis H3: Meaning in life mediates the relationship between parental autonomy support and future-oriented coping*.

### Integration of the two mechanisms

Previous studies on future time perspective and meaning in life are scarce. It is unknown whether the two variables act in parallel or serially. On the one hand, high school students adopt future-oriented coping behaviors not necessarily because they have a clear and definite perception of the future but because they are able to perceive purpose and value in their lives. From this perspective, the parallel mediating role of future time perspective and meaning in life may be present in the relationship between parental autonomy support and future-oriented coping. On the other hand, according to the socioemotional selectivity theory, individuals with higher future time perspectives have clear goals, self-perceptions, and judgments, which enable them to positively assess self-worth (Xu et al., 2021), this allows high school students to feel the meaning and value in life to an extent and to actively seek a purpose in life. In other words, the future time perspective has the functions of guidance, motivation, and direction, which can prompt individuals to think about the meaning in life. From this perspective, the serial mediating role of future time perspective and meaning in life may be present in the relationship between parental autonomy support and future-oriented coping among high school students.

Li et al. (2016) argue that parallel and serial mediations have different implications. If parallel mediation is confirmed, our interventions in either future time perspective or meaning in life help to strengthen the positive effects of parental autonomy support on future-oriented coping among high school students, and the effects of intervening in both simultaneously are more significant. If serial mediation is confirmed, we can intervene on distal factors (future time perspective) to influence the entire pathway from parental autonomy support to future-oriented coping. The existing literature does not clearly show the relationship between future time perspective and meaning in life. Therefore, we only perform an exploratory analysis of this relationship without making specific assumptions for the moment.

**Figure 1:**
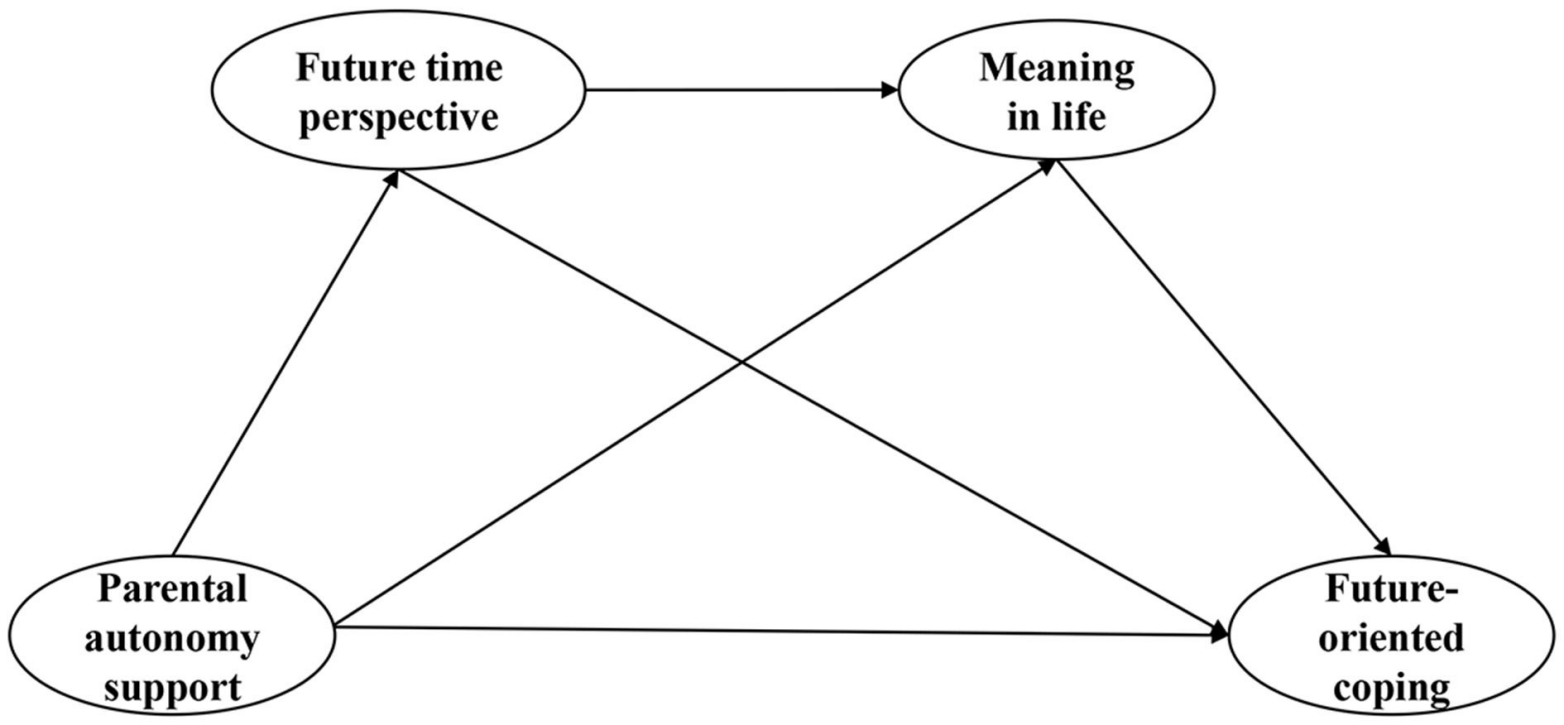
Conceptual model.

In summary, based on the ecosystem theory, self-determination theory, and social cognition theory, this study focused on the relationship between parental autonomy support and future-oriented coping among high school students and the mediating effects of future time perspective and meanings in life in it. The specific conceptual model is shown in Figure 1.

## Materials and methods

### Participants and procedures

This study was approved by the Ethics Committee of the School of Psychology, Guizhou Normal University, and the ethical approval reference number is GZNUPSY.No2021M (009). The informed consent was provided by all students. Participants were recruited from a high school located in Zunyi City, Guizhou Province, China. A total of 863 questionnaires were distributed, and after eliminating invalid questionnaires, such as incomplete information, patterned responses to questions, or too many missing values, 707 valid questionnaires were obtained. The participant effectiveness rate was 81.92%. The demographic survey results showed that the participants included 332 boys (46.96%) and 375 girls (53.04%); the age range was 15–18 years (15.95 ± 0.80); there were 506 urban students (71.57%) and 201 rural students (28.43%). There were 191 only children (27.02%) and 516 non-only children (72.98%).

### Measures

#### Parental autonomy support

Parental autonomy support was measured using the Parental Autonomy Support Scale developed by Wang et al. (2007) and translated into Chinese by Tang et al. (2013). The Chinese version has shown good reliability in measuring parental autonomy support among Chinese students (e.g., Deng et al., 2021; Peng et al., 2021). This scale has 12 items (e.g., My parents let me plan what I want to do), rated on a 5-point scale (from 1 “totally disagree” to 5 “totally agree”). Total scores for the 12 items were calculated and higher scores indicated higher levels of parental autonomy support. The Cronbach's α coefficient was 0.86.

#### Meaning in life

Meaning in life was evaluated using the Meaning in Life Scale developed by Steger et al. (2006) and revised by Wang (2013). The Chinese version has shown good reliability in measuring meaning in life among Chinese students (e.g., Wei et al., 2021; Zhang L. L. et al., 2021). The scale has 10 items across two dimensions: the presence of meaning (e.g., I understand the meaning of my life) and search for meaning (e.g., I am searching for the meaning of my life). Items were rated on a 7-point Likert scale (from 1 “totally disagree” to 7 “totally agree”). The total score of the 10 items was calculated, with higher scores indicating a stronger sense of meaning in life. The Cronbach's α coefficient was 0.71.

#### Future time perspective

Future time perspective was assessed using the Zimbardo Time Perspective Inventory (ZTPI) developed by Zimbardo and Boyd (1999) and revised by Wang (2016). The Chinese version has shown good reliability in measuring future time perspectives among Chinese residents (e.g., He et al., 2019). The scale has 5 items (e.g., I can usually complete the plan on time orderly), rated on a 5-point Likert scale (from 1 “totally disagree” to 5 “totally agree”). The total score of the 5 items was calculated and the higher the score, the stronger the future time perspective. The Cronbach's α coefficient was 0.79.

#### Future-oriented coping

Future-oriented coping was measured by the Future-Oriented Coping Scale (the Proactive Coping Inventory; PCI) developed by Greenglass et al. (1999) and revised by Gan et al. (2007). The Chinese version has shown good reliability in measuring future-oriented coping among Chinese students (e.g., Feng et al., 2015; Miao and Gan, 2021). The scale has 16 items across two dimensions: preventive coping (e.g., I will learn useful skills for future emergencies) and proactive coping (e.g., I like to overcome difficulties and meet challenges). Items were rated on a 6-point Likert scale (from 1 “totally disagree” to 6 “totally agree”). The total score of the 16 items was calculated and the higher the score, the stronger the future-oriented coping. The Cronbach's α coefficient was 0.86.

### Data analysis

SPSS 25.0 was used for common method bias tests, descriptive statistics, and correlation analysis, and Mplus 8.0 was used for structural equation model testing.

## Results

### Test for common method bias

To reduce common method bias due to participant self-report, this study controlled for it procedurally and statistically, as suggested by scholars (Zhou and Long, 2004). Procedurally, data of the four variables were collected at two-time points. Statistically, the common method bias was estimated with the Harman one-way test. The results showed that there were 9 factors with characteristic roots >1, with the first factor explaining 22.94% of the variability. This result did not exceed the 40% threshold. To ensure the reliability of the results, all items were considered as one factor, and a confirmatory factor analysis was performed. The results showed a poor model fit [χ^2^/*df* = 8.26, comparative fit index (CFI) = 0.48, Tucker-Lewis index (TLI) = 0.46, root mean square error of approximation (RMSEA) = 0.10, and standardized root mean square residual (SRMR) = 0.11]. All of the above results indicated that the common method bias was not serious in this study.

### Means, SDs, and correlations between variables

The means, SDs, and the correlation coefficient of each variable are shown in Table 1. There were significant positive correlations between parental autonomy support, meaning in life, future time perspective, and future-oriented coping.

**Table 1:** Means, SDs, and correlations between variables.

**Variables**	***M ±SD***	**1**	**2**	**3**	**4**
1 Parental autonomy support	43.40 ± 7.82	**–**			
2 Future time perspective	17.06 ± 3.14	0.24^***^	**–**		
3 Meaning in life	44.88 ± 8.73	0.22^***^	0.22^***^	–	
4 Future-oriented coping	63.41 ± 9.39	0.31^***^	0.39^***^	0.29^***^	-

*** *p < 0.001*.

### Mediation analyses

**Figure 2:**
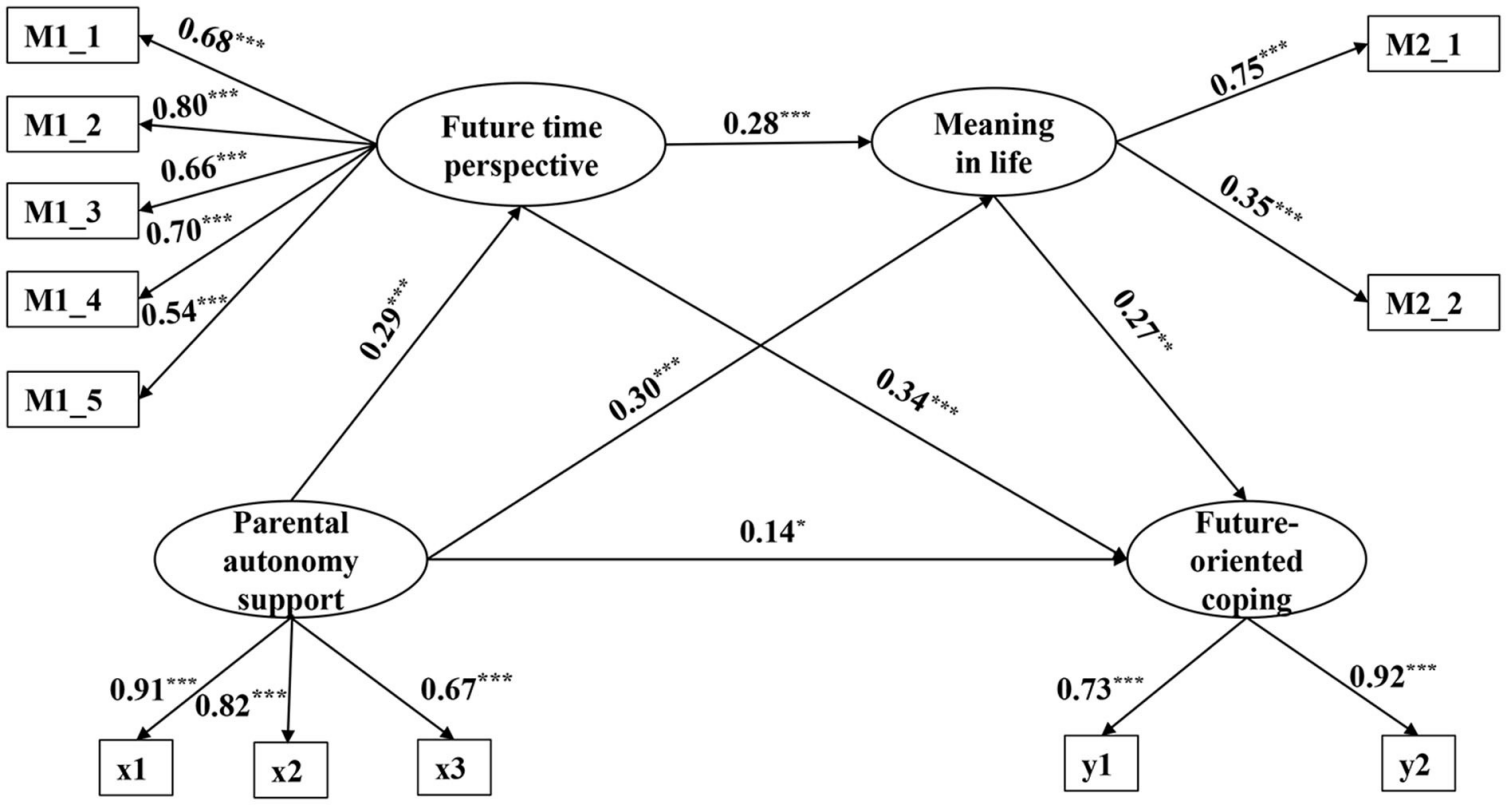
Multiple mediation effect model. *p < 0.05, **p < 0.01, ***p < 0.01.

Since there were unidimensional and multidimensional scales in the study, item parceling was applied to a unidimensional scale according to the recommendations of Wu and Wen (2011). Specifically, the contiguity method in the factorial algorithm method (i.e., packing items with similar factor loadings) was used to package the items of parental autonomy support into three indicators for structural equation modeling. Moreover, future-oriented coping was considered as a latent variable, measured by proactive coping and preventive coping in the current study (see y1 and y2 in Figure 2).

The parallel-mediated and serial-mediated models of future time perspective and meaning in life in parental autonomy support and future-oriented coping were examined by comparing the nested models. The results showed that all fit indices of the parallel and serial mediation were good (see Table 2). The two models differed significantly (Δχ^2^ = 24.62, Δ*df* = 1, *p* < 0.001). Lin and Hou (1995) believed that Δχ^2^ was significant, indicating a significant difference in the fit of the two models, in which case, the complex path (smaller *df*) was superior; The Δχ^2^ was not significant, indicating that the two models fit similarly, in which case, the simpler path (larger *df*) was superior. Therefore, the serial mediation model was the best model in this study.

**Table 2:** Parallel and chain mediation model fitting index.

	**χ^2^**	***df***	**RMSEA**	**SRMR**	**CFI**	**TLI**	**AIC**	**BIC**
Parallel mediation model	119.70	49	0.05	0.04	0.97	0.96	18,189.75	18,376.75
Chain mediation model	95.08	48	0.04	0.03	0.98	0.98	18,167.13	18,358.69

According to the Bootstrap test procedure, the data were resampled 5,000 times, enabling the size of each mediating effect to be calculated at a 95% confidence interval (CI). The results showed (see Figure 2) that parental autonomy support, future time perspective, and meaning in life all positively predicted future-oriented coping (β = 0.14, *p* < 0.05; β = 0.34, *p* < 0.001; β = 0.27, *p* < 0.01). Hypothesis 1 was verified. Parental autonomy support positively predicted future time perspective and meaning in life (β = 0.29, *p* < 0.001; β = 0.30, *p* < 0.001). Future time perspective significantly positively predicted the meaning in life (β = 0.28, *p* < 0.001). This suggests that parental autonomy support influences future-oriented coping through serial mediation of future time perspective and meaning in life.

As shown in Table 3, the mediated effect size for the path of parental autonomy support → future time perspective → future-oriented coping is 0.10. Hypothesis 2 was verified. The mediated effect size for the path of parental autonomy support → meaning in life → future-oriented coping was 0.08. Hypothesis 3 was verified. The mediated effect size for the path of parental autonomy support → future time perspective → meaning in life → future-oriented coping was 0.02. The total effect size was 0.34.

**Table 3:** The mediating effects of future time perspective and meaning in life.

	**Pathways**	**β**	**Boot SE**	**Boot LLCI**	**Boot ULCI**	**The ratio of the total effect**
Direct effect		0.14	0.06	0.03	0.25	
Indirect effect	Future time perspective	0.10	0.02	0.06	0.15	0.29
	Meaning in life	0.08	0.04	0.03	0.19	0.24
	Future time perspective and meaning in life	0.02	0.01	0.01	0.05	0.06
	Total mediation effect	0.20	0.05	0.13	0.31	0.59
Total effect		0.34	0.05	0.25	0.43	

*β refers to the standardized coefficient*.

## Discussion

The present study delineated associations between parental autonomy support and future-oriented coping, as well as the mediating role of future time perspective and meaning in life. Results showed that parental autonomy support was positively correlated with future-oriented coping. Additionally, future time perspective and meaning in life partially mediated the relationships between parental autonomy support and future-oriented coping. Further, future time perspective and meaning in life mediated the relationship between parental autonomy support and future-oriented coping sequentially.

### Theoretical implications

The study found that parental autonomy support significantly positively predicted future-oriented coping of high school students. The results are consistent with previous studies (Seiffge-Krenke and Pakalniskiene, 2011; Chen et al., 2018; Liu et al., 2019; Peng et al., 2020). This finding enriched the research on future-oriented coping and validated the unique significance of parental autonomy support in the enhancing future-oriented coping of high school students. There are several possible explanations for this finding. Firstly, as a positive parenting style, parental autonomy support creates a favorable external environment for high school students and gives them opportunities and conditions for independent growth, which facilitates high school students' ability to make independent choices and decisions, thus enhancing their ability of future-oriented coping. Secondly, parental autonomy support emphasizes appropriate moral support and psychological relief for high school students, which enhances their confidence in taking control surroundings; stimulates their internal motivation to actively plan for the future; and motivates them to adopt a positive approach to prevent or cope with potential stressors. Therefore, the more parental autonomy support, the better the future-oriented coping among high school students.

Parental autonomy support enhanced future-oriented coping through a continuous mediating role of future time perspective and meaning in life. Firstly, parental autonomy support enhances the future-oriented coping skills of high school students by improving future time perspectives. This result is similar to the findings of previous studies (Liu et al., 2009; Cheng and Wang, 2012). Parental autonomy support creates a harmonious family atmosphere for high school students. In such surroundings, high school students tend to think that the future is bright and therefore look forward to the endless possibilities of future development. At the same time, the future time perspective motivates high school students to proactively accumulate a variety of resources to enhance their future-oriented coping skills. This is in line with Pang et al.'s (2014) view of the nature of future-oriented coping as a motivational property of future time perspective.

Secondly, parental autonomy support enhances the future-oriented coping of high school students by increasing the meaning in life. This is similar to the findings of previous studies (Xiang and Hou, 2013; Zhang Y. et al., 2021). An environment of parental autonomy support can reduce the stress of high school students, enabling them to feel emotionally identified and allowing their current psychological needs to be met (Deci and Ryan, 1987). This all enhances the meaning in life for them. Increased meaning in life can motivate high school students to broaden their perspective into the future, focusing on future stress coping and establishing long-term goals.

Finally, parental autonomy support enhanced future-oriented coping through a continuous mediating role of future time perspective and meaning in life. Specifically, an environment of parental autonomy support boosts the cognitive, emotional experiences, and behavioral dispositions for the future of high school students, which enables them to maintain a more positive attitude toward the future and thus motivates them to actively pursue the meaning of life (Luo, 2021). At the same time, meaning in life as an adaptive function in the future perspective is integration across time. It enables individuals to extend their perspective from the present to the future and actively focus on future life and long-term goals, thus adopting future-oriented coping behavior (Leontiev, 2013; Roy et al., 2013). This finding clarifies the action mechanism of future time perspective and meaning in life between parental autonomy support and future-oriented coping. However, due to the low effect size of the serial mediation model, it is necessary to be cautious when applying it to explain or design intervention programs.

The present study extends the scope of research on future-oriented coping. Most previous studies have focused on past or current stressful events or assessed coping categories (e.g., positive and negative coping and approach and avoidance coping). Following the goals of positive psychology regarding the development of personal potential and the promotion of personal fulfillment, this study explored future-oriented coping and highlights its positive effects on the future life of individuals.

### Practical implications

Firstly, this study suggests that parents should encourage high school students to prepare for potential future events and provide them with appropriate assistance to reduce the negative impact of the event. Secondly, parents should guide their children to focus on the future, enhancing their awareness of the future time and planning future goals. These future goals can increase the future-oriented coping styles of high school students and keep them moving closer to their life goals. Finally, parents should strengthen life education for their children to make them feel the meaning and value in life and enhance their longing and hope for the future. Especially when negative events (such as the new crown epidemic) occur, parents need to help them actively construct meaning in life so that they can actively focus and think about the future.

## Limitations and future research

There are some limitations to this study. First, this study used cross-sectional data, which were insufficient to reveal the causal relationship between parental autonomy support and future-oriented coping. Future research could explore their relationship deeply using other methods, such as longitudinal surveys or diary methods. Second, the study sample was insufficiently representative and generalizable. Future studies could use multisource sampling. Finally, the study only tested the mediating effect of future time perspective and meaning in life, but there may be other mediating factors that could be further explored in future studies.

## Conclusion

In conclusion, this study demonstrated that parental autonomy support positively predicted future-oriented coping in high school students and also enhanced future-oriented coping through serial mediation of future time perspective and meaning in life. The findings provide a reference for enhancing the future-oriented coping of high school students.

## Data availability statement

The raw data supporting the conclusions of this article will be made available by the authors, without undue reservation.

## Ethics statement

This study involving human participants was reviewed and approved by the Ethics Committee of the School of Psychology, Guizhou Normal University. The participants provided their written informed consent to participate in this study. Written informed consent to participate in this study was provided by the participants' legal guardian/next of kin.

## Author contributions

LZ, XP, XZ, and HW were in charge of literature review, data collection, data analysis, and writing. LZ, SX, and YC ensured supervision over selecting the topic and research design. All authors contributed to the article and approved the submitted version.

## Funding

This study was supported by the Postgraduate Education Innovation Program of Guizhou Province, Qianjiaohe (No. YJSKYJJ[2021]108).

## Conflict of interest

The authors declare that the research was conducted in the absence of any commercial or financial relationships that could be construed as a potential conflict of interest.

## Publisher's note

All claims expressed in this article are solely those of the authors and do not necessarily represent those of their affiliated organizations, or those of the publisher, the editors and the reviewers. Any product that may be evaluated in this article, or claim that may be made by its manufacturer, is not guaranteed or endorsed by the publisher.

## References

[B1] Bandura A. (1986). Social Foundations of Thought and Action: A Social Cognitive Theory. Upper Saddle River, NJ: Prentice-Hal, Inc.

[B2] Bronfenbrenner U. (1986). Ecology of the family as a context for human development: research perspectives. Dev. Psychol. 22, 723–742. 10.1037/0012-1649.22.6.723

[B3] Chen Q. Sang Y. Y. Wang H. Yang T. Z. (2018). Mediating effect of coping style between parenting styles and anxiety in junior high school students. Chin. J. Health Stat. 35, 843–845.

[B4] Cheng Z. H. Wang Y. R. (2012). On the relationship between parental rearing patterns and future time perspective of university students. J. Beibu Gulf Univ. 27, 66–68. 10.3969/j.issn.1673-8314.2012.06.016

[B5] Deci E. L. Ryan R. M. (1987). The support of autonomy and the control of behavior. J Person. Soc. Psychol. 53, 1024–1037. 10.1037/0022-3514.53.6.1024 3320334

[B6] Deci E. L. Ryan R. M. (2008). Facilitating optimal motivation and psychological well-being across life's domains. Can. Psychol. Psychol. Canad. 49, 14–23. 10.1037/0708-5591.49.1.14

[B7] Deng L. Y. Liu X. T. Tang Y. Q. Yang M. X. Li B. L. (2021). Parental psychological control, autonomous support and adolescent internet gaming disorder: the mediating role of impulsivity. *Chin J. Clin. Psychol*. 29, 316–322. 10.16128/j.cnki.1005-3611.2021.02.020

[B8] Feng S. H. Huang X. T. (2002). Proactive coping: a kind of future-oriented coping. Expl. Psychol. 22, 31–35. 10.3969/j.issn.1003-5184.2002.02.007

[B9] Feng Y. Gan Y. Q. Liu Z. X. Nie H. Y. Chen W. Y. (2015). Relationship between uncertainty response and future-oriented coping: the mediation of anticipated emotion. Acta Sci. Nat. Univ. Pekin. 51, 485–494. 10.13209/j.0479-8023.2015.025

[B10] Gan Y. Q. (2011). The sequential model of future-oriented coping and its time perspective mechanism. Adv. Psychol. Sci. 19, 1583–1587. 10.3724/SP.J.1042.2011.01583

[B11] Gan Y. Q. Yang M. S. Zhou Y. Zhang Y. L. (2007). The two-factor structure of future-oriented coping and its mediating role in student engagement. Person. Indiv. Differen. 43, 851–863. 10.1016/j.paid.2007.02.009

[B12] Greenglass E. Schwarzer R. Jakubiec S. D. Fiksenbaum L. Taubert S. (1999). “The proactive coping inventory (PCI): a multidimensional research instrument,” in Paper presented at the 20th International Conference of the STAR (Stress and Anxi-ety Research Society) (Cracow) 12–14.

[B13] He W. F. Lu H. C. Du G. (2019). The relationship between two fundamental dimensions and subjective well-being: the mediation effect of future time perspective. J. Psychol. Sci. 42, 1167–1173. 10.16719/j.cnki.1671-6981.20190521

[B14] Lambert N. M. Stillman T. F. Hicks J. A. Kamble S. Baumeister R. F. Fincham F. D. (2013). To belong is to matter: sense of belonging enhances meaning in life. Personal. Soc. Psychol. Bull. 39, 1418–1427. 10.1177/0146167213499186 23950557

[B15] Leontiev D. A. (2013). Personal meaning: a challenge for psychology. J. Posit. Psychol. 8, 459–470. 10.1080/17439760.2013.830767

[B16] Li D. P. Zhou Y. Y. Zhao L. Y. Wang Y. H. Sun W. Q. (2016). Cumulative ecological risk and adolescent Internet addiction: the mediating role of basic psychological need satisfaction and positive outcome expectancy. Acta Psychol. Sin. 48, 1519–1537. 10.3724/SP.J.1041.2016.01519

[B17] Lin W. Y. Hou J. T. (1995). Structural equation analysis-model equivalence and modification. J. Educ. Stud. 23, 147–162.

[B18] Liu H. Zhuo Y. L. Sun J. Wei M. Zhang X. X. Li Y. (2019). Parental rearing patterns and coping styles on undergraduate students' anxiety. Mod. Prev. Med. 46, 1635–1638.

[B19] Liu X. F. Zheng Y. Li C. J. (2009). Relationship between time perspective coping style and health among undergraduates. Chin. J. School Health. 30, 533–534.

[B20] Liu X. X. Gong S. Y. Zhou Z. J. Feng X. W. Yu Q. L. (2020). The relationship among parental autonomy support, parental psychological control, and junior high school students' creative self-efficacy: the mediating role of academic emotions. Psychol. Dev. Educ. 36, 45–53. 10.16187/j.cnki.issn1001-4918.2020.01.06

[B21] Luo F. J. (2021). The influence of future time perspective on career adaptation in high school students: meaning in life and the chain mediating effect of psychological capital (Master Thesis). Chengdu: Sichuan Normal University.

[B22] Luo J. Cui H. Q. Dai X. Y. Zhao S. Y. (2014). The relationship between the social support of upper secondary school students and their coping styles: the mediating effect of self-efficacy. Chin. J. Spec. Educ. 92–96. 10.3969/j.issn.1007-3728.2014.10.016

[B23] Luo X. M. He H. (2020). The influence of parental autonomy support and meaning in life on the academic self-efficacy of junior high school students. Mental Health Educ. Prim. Seco. School. 20, 18–21+24. 10.3969/j.issn.1671-2684.2020.21.005

[B24] Miao M. Gan Y. Q. (2018). The promotional role of meaning in life in future-oriented coping: positive affect as a mediator. Int. J. Psychol. 55, 52–59. 10.1002/ijop.12543 30362105

[B25] Miao M. Gan Y. Q. (2021). The predictive effects of presence of meaning in life on future-oriented coping: the chain mediating role of positive affect and resource accumulation. Stud. Psychol. Behav. 19, 403–409. 10.3969/j.issn.1672-0628.2021.03.017

[B26] Miao M. Wang Y. Q. Ye Q. Ke Q. Gan Y. Q. (2017a). Verification of the sequential model of future- oriented coping in engaged individuals. Chin. J. Clin. Psychol. 25, 678–683. 10.16128/j.cnki.1005-3611.2017.04.020

[B27] Miao M. Zheng L. Gan Y. Q. (2017b). Meaning in life promotes proactive coping via positive affect: a daily diary study. J. Happin. Stud. 18, 1683–1696. 10.1007/s10902-016-9791-4

[B28] Miao M. Zheng L. Gan Y. Q. (2021). Future-oriented function of meaning in life: Promoting hope via future temporal focus. Person. Indiv. Differen. 179, 1–8. 10.1016/j.paid.2021.110897

[B29] Pang X. Lv H. C. Hua S. X. (2014). Delay of gratification: self- regulation based on the future time perspective. J. Psychol. Sci. 37 78–82. 10.16719/j.cnki.1671-6981.2014.01.015

[B30] Peng S. Niu G. F. Wang X. Zhang H. P. Hu X. E. (2021). Parental autonomy support and adolescents' positive emotional adjustment: mediating and moderating roles of basic need satisfaction. Psychol. Dev. Educ. 37, 240–248. 10.16187/j.cnki.issn1001-4918.2021.02.11

[B31] Peng Z. F. Fu N. Zhang X. J. (2020). The relationship between interparental conflict and middle school students' coping style: the chain mediating effect of parental rearing style and emotional security. Psychol. Dev. Educ. 36, 668–676. 10.16187/j.cnki.issn1001-4918.2020.06.04

[B32] Próchniak P. Próchniak A. (2021). Future-oriented coping with weather stress among mountain hikers: temperamental personality predictors and profiles. Behav. Sci. 11, 1–13. 10.3390/bs11020015 33498900 PMC7912429

[B33] Roy F. Baumeister K. D. Vohs J. L. Aaker E. (2013). Some key differences between a happy life and a meaningful life. J. Posit. Psychol. 8, 505–516. 10.1080/17439760.2013.830764

[B34] Ryan R. M. Deci E. L. (2000). Self-determination theory and the facilitation of intrinsic motivation, social development, and wellbeing. Am. Psychol. 55, 68–78. 10.1037/0003-066X.55.1.68 11392867

[B35] Ryan R. M. Deci E. L. Grolnick W. S. Guardia J. (2015). The Significance of Autonomy and Autonomy Support in Psychological Development and Psychopathology. Hoboken, NJ: John Wiley & Sons, Ltd.

[B36] Schwarzer R. Luszczynska A. (2008). Reactive, anticipatory, preventive, and proactive coping: a theoretical distinction. Prev. Res. 15, 22–24.

[B37] Schwarzer R. Taubert S. (2002). “Tenacious goal pursuits and striving toward personal growth: proactive coping,” in Beyond Coping: Meeting goals, Visions, and Challenges, eds E. Fydenberg (London: Oxford University) 19–35.

[B38] Seiffge-Krenke I. Pakalniskiene V. (2011). Who shapes whom in the family: reciprocal links between autonomy support in the family and parents' and adolescents' coping behaviors. J. Youth Adol. 40, 983–995. 10.1007/s10964-010-9603-9 21107666

[B39] Shek D. Chai C. Dou D. (2021). Parenting factors and meaning in life among Chinese adolescents: a six-wave longitudinal study. J. Adol. 87, 117–132. 10.1016/j.adolescence.2021.01.004 33581398

[B40] Song G. W. Bao W. J. He G. W. (2013). Learning burnout and its relationship with future time perspective and achievement goal orientation among middle school students. Stud. Psychol. Behav. 11, 478–482. 10.3969/j.issn.1672-0628.2013.04.008

[B41] Sougleris C. Ranzijn R. (2011). Proactive coping in community-dwelling older Australians. Int. J. Aging. Human Dev. 72, 155–168. 10.2190/AG.72.2.d 21639015

[B42] Steger M. F. Frazier P. Oishi S. Kaler M. (2006). The meaning in life questionnaire: assessing the presence of and search for meaning in life. J. Coun. Psychol. 53, 80–93. 10.1037/0022-0167.53.1.80

[B43] Steger M. F. Kawabata Y. Shimai S. Otake K. (2008). The meaningful life in Japan and the United States: levels and correlates of meaning in life. J. Res. Person. 42, 660–678. 10.1016/j.jrp.2007.09.003

[B44] Tang Q. Fang X. Y. Hu W. Chen H. D. Wu M. X. Wang F. (2013). The associations between parental and teacher autonomy support and high school students' development. Psychol. Dev. Educ. 29, 604–615. 10.16187/j.cnki.issn1001-4918.2013.06.003

[B45] Wang C. (2016). Time perspective: the revision of the inventory and the influence on risky driving behavior (Master Thesis). Chongqing: Southwest University.

[B46] Wang Q. Pomerantz E. M. Chen H. C. (2007). The role of parents' control inearly adolescents' psychological functioning: a longitudinal investigation in the United States and China. Child Dev. 78, 1592–1610. 10.1111/j.1467-8624.2007.01085.x 17883450

[B47] Wang X. Q. (2013). Psychometric evaluation of the meaning in life questionnaire in Chinese middle school students. Chin. J. Clin. Psychol. 21, 764–767. 10.16128/j.cnki.1005-3611.2013.05.008

[B48] Wei L. Z. Liu Y. L. Liu C. X. Lin J. Wang X. (2021). The effect of family cohesion on mental health of high school students:a moderated mediation model. Stud. Psychol. Behav. 19, 361–367. 10.3969/j.issn.1672-0628.2021.03.01135502310

[B49] Weinstein N. Ryan W. S. Dehaan C. R. Przybylski A. K. Legate N. Ryan R. M. (2012). Parental autonomy support and discrepancies between implicit and explicit sexual identities: Dynamics of self-acceptance and defense. J. Person. Soc. Psychol. 102, 815–832. 10.1037/a0026854 22288529

[B50] Wen M. Gan Y. Q. Jiang H. F. Du W. W. Yang X. R. Chen Y. T.. (2014). From achievement motivation to academic burnout and engagement: longitudinal mediating effect of future-oriented coping. Acta Sci. Nat. Univ. Pekin. 50, 388–396. 10.13209/j.0479-8023.2014.036

[B51] Wu Y. Wen Z. L. (2011). Item parceling strategies in structural equation modeling. Adv. Psychol. Sci. 19, 1859–1867. 10.3724/SP.J.1042.2011.01859

[B52] Xiang M. Q. Hou X. H. (2013). The relationship between the social support of hearing-impaired middle school students and their sense of the meaning in life: the mediating effect of core self-evaluation. Chin. J. Spec. Educ. 20, 34–40. 10.3969/j.issn.1007-3728.2013.06.007

[B53] Xu B. B. Chen X. Y. Wang J. Y. Li J. J. (2021). Future time perspective and career decision-making self-efficacy: chain mediating effect of perceived social support and self-esteem. Psychol. Exp. 41, 276–281+288.

[B54] Yu X. X. Deng L. M. (2019). Relationship between social support and meaning in life in rural left-behind junior high school students: the mediating role of optimism. Mod. Prev. Med. 46, 2807–2809.

[B55] Zhang L. L. Xu J. Zhu A. H. Xie L. Li Y. L. (2021). Influence of perceived social support on college students' coping style: Chain mediating effect of meaning in life and psychological resilience. China J. Health Psychol. 29, 758–761. 10.13342/j.cnki.cjhp.2021.05.024

[B56] Zhang Y. Yuan B. Wang K. Shen T. (2021). The effect of impulsivity traits on senior high school students' suicidal ideation: the role of campus exclusion and sense of life meaning. Stud. Psychol. Behav. 19, 85–95.

[B57] Zhou H. Long L. R. (2004). Statistical remedies for common method biases. Adv. Psychol. Sci. 12, 942–950. 10.3969/j.issn.1671-3710.2004.06.018

[B58] Zimbardo P. G. Boyd J. N. (1999). Putting time in perspective: a valid, reliable individual differences metric. J. Person. Soc. Psychol. 77, 1271–1288. 10.1037/0022-3514.77.6.1271

